# The Braking-Pressure and Driving-Direction Determination System (BDDS) Using Road Roughness and Passenger Conditions of Surrounding Vehicles

**DOI:** 10.3390/s22124414

**Published:** 2022-06-10

**Authors:** YiNa Jeong, SuRak Son, ByungKwan Lee, SuHee Lee

**Affiliations:** 1 Department of Software, College of Engineering, Catholic Kwandong University, Gangneung 210-701, Korea; lupinus07@nate.com (Y.J.); sonsur@naver.com (S.S.); bklee@cku.ac.kr (B.L.); 2 Department of Beauty Design, College of Media & Art, Catholic Kwandong University, Gangneung 210-701, Korea

**Keywords:** roughness classification, K-nearest neighbor, braking pressure, steering angle, convolutional neural network, AI and deep learning, control, optimization, prediction

## Abstract

A fully autonomous vehicle must ensure not only fully autonomous driving but also the safety and comfort of its passengers. However, the self-driving technology that is currently completed focuses only on perfect driving and does not guarantee the safety and comfort of passengers. This paper proposes a braking-pressure and driving-direction determination system (BDDS), which computes the brake pressure and steering angle optimized for passenger safety by utilizing more diverse information than existing autonomous vehicles. The BDDS proposed in this paper consists of two modules. The road roughness classification module (RRCM) classifies the roughness of the road by using the pressure data applied to the suspension and the K-NN algorithm and computes the optimal brake pressure. The passenger recognition and sharing module (PRSM) identifies the current occupant status of the vehicle by using a body pressure sensor and CNN, shares the information with surrounding vehicles, and computes the optimal steering angle using passenger information and road information. As a result of the simulations described in this paper, the parameters of AI models were optimized. In addition, the RRCS was about 7% more accurate than the K-means clustering algorithm, and PRS was about 9% more accurate than the existing seat recognition system.

## 1. Introduction

Self-driving cars have made a leap in development thanks to the efforts of companies such as Google and Tesla. The typical use of autonomous vehicles for widespread use on public roads is likely to be possible in a few years, but these vehicles are already being used in “constrained” applications such as open pit mining and agriculture. Among the many technologies that enable autonomous vehicles are a combination of sensors and actuators, sophisticated algorithms, and powerful processors that run software [1]. With advances in autonomous vehicle testing from companies such as Google, Apple (in USA), Tesla (in Toronto, Brooklyn etc.), Uber, and Lyft (in USA), the first signs of a driverless future were seen [2].

Autonomous vehicles respond to various situations by using the vehicle’s electronic control unit (ECU), actuators, and various sensors. In addition, the autonomous vehicle communicates not only with the vehicle itself but also with surrounding vehicles and road-side unit (RSU) through cellular networks, Wi-Fi, etc. Through this communication, the current autonomous vehicles can detect a crisis (for example, another vehicle approaching suddenly in the vicinity, a pedestrian, icy road, etc.) and send an alert to the user. These vehicles may also be configured with mechanisms that take active steps to avoid these hazards [3].

After the completion of Level 3 autonomous driving technology, an autonomous driving technology for Levels 4 and 5 must now ensure not only the driving situation but also the comfort of the passengers. However, current autonomous driving technology cannot fully guarantee a passenger’s comfort. To ensure passenger comfort, it is necessary to use several items of data as well as visual information currently being collected (LiDAR, front camera). Therefore, this work aimed to calculate the optimal braking pressure and steering angle while driving a vehicle using the data on the seat pressure inside the vehicle and the pressure data on the suspension.

This paper proposes the braking-pressure and driving-direction determination system (BDDS). The BDDS collects pressure data from the vehicle’s suspension to classify the roughness of the road and accurately identifies the passenger status of itself and surrounding vehicles through body pressure sensors and V2V. Next, The BDDS uses road roughness, passenger conditions, driving information, and weather information to determine the optimal brake pressure strength and steering angle. The BDDS consists of the road roughness classification module (RRCM), which determines the roughness of the current road by receiving the pressure of the three axes as frequencies from the vehicle’s suspension; the passenger recognition and sharing module (PRSM) to classify the condition of passengers inside the vehicle into luggage, children, and adults by using a body pressure sensor, determine what it is, and then share the information with surrounding vehicles; and the brake pressure and direction determination module (BPDDM), which uses the output of RRCM and PRSM to determine the optimal brake pressure and steering angle.

The composition of this paper is as follows. Section 2 describes the existing studies related to the passengers and road roughness in this paper. Section 3 details the structure and operation of the BDDS. Section 4 compares it with the existing methods to analyze the performance. Section 5 discusses the conclusion of the proposed BDDS and future research directions.

## 2. Related Works

To improve the overall vehicle as well as the autonomous driving, research on passengers and road statements have been continuously conducted. This section introduces several studies of passengers and road statements and describes the approach the BDDS takes to analyze passengers and road statements.

### 2.1. Passengers of an Autonomous Vehicles

Most of the research on passengers is focused on ensuring the safety of passengers. Yuxi Guo et al. evaluated the effects of independently designed vehicle driving condition prompt (DCP) systems on subjective passenger comfort and motion sickness [4]. Before performing the obstacle avoidance maneuver, the DCP system informed the passenger of “obstacle avoidance forward” and “deceleration forward” before the deceleration maneuver. This study demonstrated that passengers’ subjective comfort was improved when riding with the DCP system.

Petri Launonen et al. investigated whether the attitude of passengers changed in an icy road environment [5]. The study found that trust, safety, and security were the main factors influencing people’s positive attitude toward the use of autonomous vehicles. Results for passengers traveling in harsh winter conditions indicated that winter conditions do not significantly affect passenger attitudes toward autonomous vehicle use.

Chongfeng Wei et al. evaluated the tolerance for lateral offsets based on the measured driving performance of real drivers and their responses to various risk factors [6]. The purpose of this study was to prevent collisions between vehicles and other road users and road edges, as well as provide stability and comfort to drivers. The experimental results of this study proved that a vehicle trajectory generation that can ensure the driver’s comfort on the road is possible through the offsets’ setting.

Sarah Atifah Saruchi et al. proposed the utilization of a fuzzy-proportional integral derivative (PID) controller for a motion sickness (MS) minimization control structure. In this study, the interaction of the lateral acceleration and head tilt concept was adopted to diminish the lateral acceleration [7]. This study aimed to minimize motion sickness through the controller of an autonomous vehicle. The head movement was used as the controlled variable to compute the corrective wheel angle. The results showed that the averages of motion sickness incidence (MSI) index can be lowered by 3.95% for single lap and 11.49% for 10 laps.

Yuli Zhao et al. reviewed the recent developments in active seat suspensions for vehicles [8]. This study and the methods for adjusting the stability of the vehicle seats were studied, and future research directions were suggested. According to this study, compared to conventional controllers, an artificial neural network (ANN) control reduces many modeling and computation steps and simplifies controller design. However, the ANN training required a large amount of ideal data for backpropagation training. Therefore, this study proposed combining the ANN system with the existing control system and using the control results of the existing system to train the ANN controller and increase the robustness of the system.

Guoqing Geng et al. proposed a route planning method that mimics the lane change behavior of good drivers for passenger comfort and stability [9]. This study designed a lane change model by training a genetic algorithm (GA) and backpropagation (BP) neural network with lane change data from good drivers. As a result of the simulation, it was confirmed that the route generated by the lane change model proposed in this study was the same as that traveled by an excellent driver under the same conditions. In other words, autonomous vehicles can reflect the driving habits of good drivers.

Abu Saleh Md Bakibilah et al. proposed an incentive-based, dynamic ride-sharing transportation system for smart cities that can share empty seats in privately owned vehicles [10]. The proposed system aimed to reduce the number of private vehicles in order to solve the severe traffic congestion occurring in the city. Dynamic ridesharing operates on a mobile cloud-based system that matches drivers and rides in real time.

Alexandros Leledakis et al. aimed to investigate the effect of various postures of car front-seat passengers on the kinematic response when a collision occurs at an intersection [11]. This study quantified the posture of the occupant using a cross-correlation method to investigate the effect of occupant posture under various impact conditions. Experimental results from that study demonstrated that, in a crash, forward leaning poses more problems for occupant protection than moderately back leaning.

Pier Giuseppe Anselma proposed a multi-target, off-line optimal control method for estimating the speed of a following vehicle in adaptive cruise control (ACC) [12]. This implemented method relies on the principle of global optimality using dynamic programming and focuses on improving passenger comfort by minimizing the vehicle’s energy consumption. As a result of the experiment in this study, it was possible to confirm an energy saving of 22% and an increase in passenger convenience by 48% in a vehicle that supports ACC applying this method compared to a conventional vehicle.

As the interior of a vehicle is a confined space, Ju Yeong Kwon et al. designed a flexible seat layout based on the total space of the vehicle [13]. Since the driver is no longer present in autonomous vehicles, this study placed the face-to-face seats to make the most of the vehicle’s space and analyzed the relationship between various factors related to space. Using mock-up, observational surveys, questionnaires, and the think-aloud research method within an ethnographic observation framework, they conducted experiments on three study participants who were aware of the changing concept of autonomous vehicles. The main result of this study is that a face-to-face seating arrangement generates various activities of passengers. Therefore, the design of space components based on a face-to-face seating arrangement only considers the activities of the passengers.

Current studies analyze the driving status of a vehicle, focusing on energy saving, passengers’ subjective comfort (prevention of motion sickness), and passenger safety based on passenger conditions. Therefore, the BDDS of this paper focuses on calculating the optimal steering angle according to the passenger’s condition in an autonomous vehicle, excluding environmental factors to ensure passenger safety and prevent vehicle accidents.

### 2.2. Road Roughness Classification of Autonomous Vehicles

The classification of road roughness has attracted attention as a very important research topic after the commercialization of Level 3 autonomous driving vehicles. The effect of the road roughness on tires and suspension must be analyzed in order to determine the braking method of a fully autonomous vehicle. The following are studies on the roughness of roads. H.M. Ngwangwa et al. reported the performance of an artificial neural network-based road condition monitoring methodology on measured data obtained from a Land Rover Defender 110, which was driven over discrete obstacles and Belgian paving [14]. The neural network in this study was trained on data computed by Land Rover’s numerical model. As a result of the experiment in this study, the model trained by dissolving the Land Rover data showed better quality than the existing handling mode. However, it was observed that the overall network performance for bumps was impaired due to the poor roll motion estimation capability of the numerical model and the use of the point-follower tire model.

Shahram Misaghi et al. presented a process for quantifying the effects of truck suspension systems and road surface conditions on pavement damage [15]. The international roughness index (IRI) was used to simulate road roughness. Additionally, the interaction between the truck and the pavement was modeled to calculate the load on the pavement. The main purpose of this study was to present a method by which the interaction between the road and the vehicle can evaluate the performance of the road pavement. After a parametric study, it was found that the spring strength of the suspension and the roughness of the road surface affect the impact of the vehicle. As the vehicle speed increased, the dynamic impact increased significantly. Moreover, the suspension damping factor had a significant impact on the vehicle’s speed and diesel direct injection.

Mohammad Arbabpour Bidgoli et al. aimed to develop a new, automated, cost-effective data collection device for measuring roughness [16]. In this study, a cost-effective road roughness monitoring system (RRMS) was developed by mounting an additional accelerometer on the wheel of a vehicle, and the roughness of the pavement was measured using the RRMS. As a result of the experiment, when calculating the roughness of the road, the error of the proposed system was less than 10% in RMSE and less than 20% in the average error percentage.

Konstantina Georgouli et al. presented and discussed currently published relevant research and findings on the quantification of the wheel wander, both on the negative and on the positive aspects, and provided potential insightful future areas of enquiry to help mold and shape future research for this emerging field [17]. This study focused on all the effects of zero lateral wheel wander of autonomous vehicles on the accumulated damage on new and existing pavements. As a result of the study, it was proven that autonomous vehicles using zero wheel wander tend to keep driving in the center of the lane, so the lifespan of the road is shortened quickly and the damage to the road is accumulated more.

Kevin Herubiel Floreán-Aquino et al. described semi-active modern control schemes for a quarter-vehicle suspension with a magnetorheological damper (MRD) to attenuate vibrations and simultaneously improve the passenger comfort and the vehicle road holding [18]. The proposed control schemes were compared with a traditional sky-hook semi-active control, showing a considerable improvement in the closed-loop system performance at the two main vibration modes simultaneously.

Changbai Wang et al. developed an AdaBoost regression (ABR) model to improve the predictive performance of road roughness and compared it with the linear regression model of the existing Mechanistic-Empirical Pavement Design Guide (MEPDG) [19]. As a result, the correlation coefficient (R2) between the measured and predicted values of the test set increased from 0.5118 to 0.9751, and the mean squared error (MSE) decreased from 0.0245 to 0.0088. Therefore, this study showed that the ABR model is much better than the LR. The LR was found to be unable to analyze many additional deterministic factors, such as raveling and bleeding, that affect the international roughness index (IRI).

Vidas Žuraulis et al. proposed that the existing pavement quality evaluation indexes be analyzed and a methodology for adapting them for roads with gravel pavement be created [20]. The goal of this study was to develop the methodology for gravel road quality evaluation concerning pavement deterioration and vehicle dynamic response, providing an appropriate maintenance indication for this pavement type. In this study, the roughness of gravel pavement 160 m was measured three times on different environments using the 3 m straightedge method. The first condition was after 2 days of road blading, the second condition was after 3 weeks of pavement maintenance, and the third condition was the worst condition before maintenance. Measurements have shown that the rougher the pavement, the sharper the quality of the road.

Vaidas Lukoševičius et al. examined the vehicle stability dynamic models and develop a refined methodology for estimating the key parameters of these models [21]. Additionally, this study produced a revised tire smoothing function model that takes into account tread deflections. Finally, they investigated vertical dynamics models and develop recommendations for their use in vehicle stability studies.

Aurimas Čerškus et al. proposed a method to detect the profile characteristics of a road using an acceleration value that can be easily measured using a mount sensor [22]. This study estimated the spectral density of the road profile for wheel hub acceleration using a transfer function. The power spectral density (PSD) allows for the adjustment of a vehicle driving mode in the case of driver assistant systems or fully automated driving mode, which sets damping parameters for adjustable shock absorbers or provides driving hints for the driver.

Georgios Papaioannou et al. aimed to minimize tire wear, ensure occupant comfort, and optimize parameters to improve vehicle handling [23]. This study used optimization as a tool to identify and highlight the balancing relationships between various parameters. Therefore, parameters were applied to specific tires, optimized for the vehicle while cornering on different Classes A and B road roughness profiles. In this study, comfort illustrates the same conflicting relation with wear as with vehicle stability.

Qingxia Zhang et al. proposed a road roughness estimation method using the frequency response function (FRF) of a vehicle [24]. In this study, the equation for the vehicle response, road roughness, and vehicle FRF was deduced and set up in a linear equation system; hence, road roughness can be estimated using the vehicle FRF and the measured vehicle responses. The vehicle FRF can be calculated by a direct estimation of the measured vehicle accelerations using the least squares method. Moreover, the shape function method can be used to eliminate the singular and noisy parts of the estimated FRF and to improve the accuracy of the estimated road roughness profile. The road roughness can be estimated online with a few seconds of time delay.

As above, current studies analyze the effect of road roughness on tires and suspension to make autonomous vehicles drive optimally. Therefore, the BDDS proposed in this study classifies the road roughness in detail for fully autonomous driving and calculates the optimal brake pressure based on the road roughness and road information.

## 3. A Design of the Braking-Pressure and Driving-Direction Determination System (BDDS)

### 3.1. Overview

Currently, autonomous vehicles are being commercialized after testing. However, although autonomous vehicles have not yet been fully commercialized, 81 accidents have occurred; the driving method of vehicles to avoid accidents relies heavily on LiDAR. In addition, most studies to avoid them are conducting experiments using limited conditions. In order to achieve Level 4 autonomous vehicles, the environment an autonomous vehicle can perceive must be expanded, which must influence driving decisions. Therefore, this paper proposes the braking-pressure and driving-direction determination system (BDDS) using road roughness and passenger conditions of surrounding vehicles to improve the driving stability of autonomous vehicles. The BDDS accurately recognizes the roughness of the road on which the vehicle is driving, including road information and weather information, which are pieces of information collected from existing autonomous vehicles, as well as the condition of the vehicle itself, its passengers, and passengers in surrounding vehicles and determines the vehicle’s driving direction so that it can maintain optimal driving and brake pressure.

**Figure 1:**
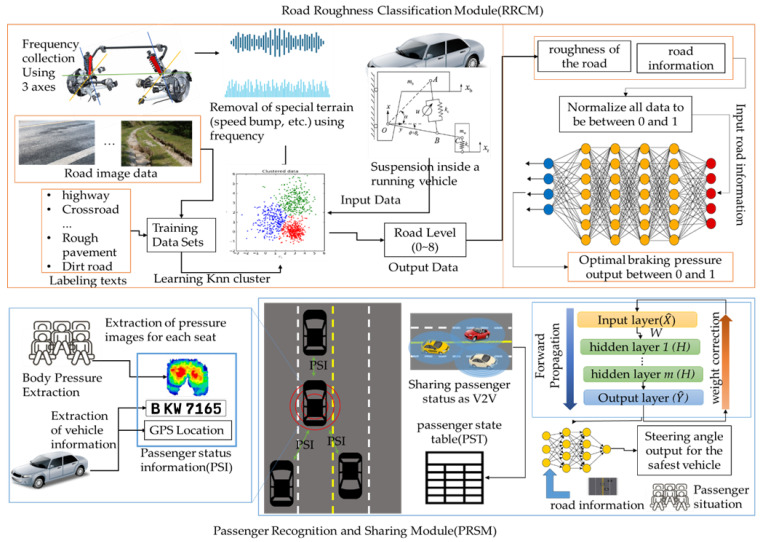
The structure of a BDDS.

The BDDS consists of two modules, as shown in Figure 1. The road roughness classification module (RRCM) collects the pressure of the wheels while driving on the road by installing three axles on the vehicle’s suspension and classifies the roughness of the road on which the vehicle is traveling into eight levels by using the pressure. It also calculates the best brake pressure for the current road environment by using the roughness of the road and the speed of the surrounding vehicles. The passenger recognition and sharing module (PRSM) classifies the passenger status of a vehicle more accurately using a body pressure sensor. The PRSM recognizes whether an adult, child, or cargo is seated on each seat of the vehicle and stores the recognized passenger status in passenger status information (PSI) along with the vehicle’s location and vehicle number. By sharing the PSI with surrounding vehicles through V2V, the vehicle can accurately recognize the location of nearby vehicles and the status of passengers. The PRSM calculates the steering angle at which the vehicle can safely travel to its destination based on the position of the surrounding vehicles and the condition of the passengers.

### 3.2. The Road Roughness Classification Module (RRCM)

**Figure 2:**
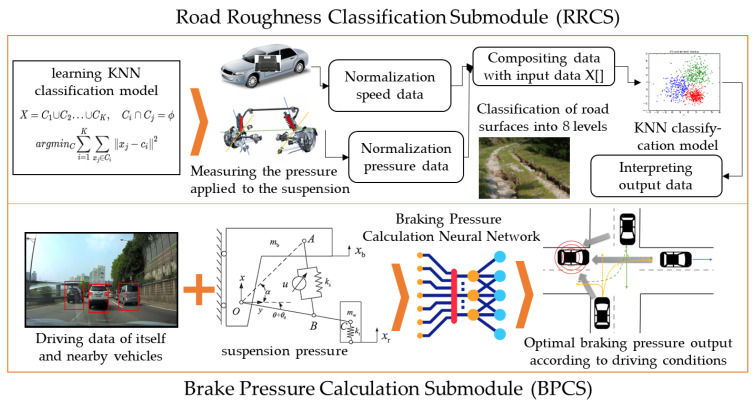
The structure of the RRCM.

The road roughness affects various aspects of driving, such as the speed of the vehicle, the load on the suspension system, and the degree of vibration felt by passengers [25,26,27]. Knowing the condition of the road is important. Whether the road is wet or bumpy has a big impact on the vehicle’s braking distance. Especially in emergency situations, the vehicle must calculate the appropriate braking pressure according to the road conditions. Therefore, road roughness has not been considered in existing autonomous vehicles that focus on avoiding obstacles but must be considered in fully autonomous vehicles that consider both passenger condition and vehicle stability. In this work, the road roughness classification module (RRCM) was designed to calculate the optimal braking pressure in order not to lose the tire grip in various road conditions. The RRCM consists of two submodules. First, the road roughness classification submodule (RRCS) classifies the roughness of the road on which the vehicle is traveling into eight levels through K-nearest neighbor (KNN) clustering, which is one of the machine learning techniques [28,29]. Second, the brake pressure calculation submodule (BPCS) calculates the most suitable brake pressure for driving using the roughness of the road classified by the RRCS, the condition of surrounding vehicles, and the current road condition. Figure 2 shows the overall structure of the RRCM.

#### 3.2.1. A Design of the RRCS

The RRCS proposed in this paper accurately classifies the roughness of the road on the driving vehicle through four stages. First, the KNN algorithm is learned using the training data set scaled between 0 and 1. Because the RRCS uses eight clusters, it uses a KNN algorithm capable of supervised learning, not K-means in which the initial center point is arbitrarily set. For the KNN to be classified correctly, the normalized input data set, the labels for training, and the number of K must be determined. The RRCS scales the data between 0 and 1 using z-score normalization to remove an abnormal value among pressure data appearing on the road. However, in the case of data that are not collected numerically, such as “Is there a speed bump?”, they are converted into a Boolean variable using one hot encoding. The K number for the KNN is determined through K-fold cross validation.

(1) $$x_{i}^{\prime }=\frac{x_{i}-m}{\delta },$$

Equation (1) represents z-score normalization. In Equation (1), *x_i_* denotes the collected data such as in cs and a in Table 1, *m* denotes the average value of all data, and *δ* denotes the standard deviation of all data. Table 1 shows the input values of the training data and the labels of the classified clusters used for the RRCS.

Second, the RRCS calculates the pressure applied to each wheel while driving by installing four three-axes pressure sensors on the suspension for accurate road roughness analysis. Of the three axes, the vertical axis (*y* axis) is the same as the frequency data of the suspension collected from a general vehicle. The *y* axis ranges from 0.5 to 2.0 Hz and measures how much the vehicle is lifted up and down. the horizontal axis (*x* axis) means the left and right pressure of the vehicle. The RRCS sets the vehicle’s standstill state to 0 Hz on the *x* axis and measures the frequency between −2.0 and 2.0 Hz to indicate which direction the vehicle is leaning, to the left or right. The other horizontal axis (*z* axis) is the front and rear pressure of the vehicle. The RRCS sets the vehicle’s standstill state to 0 Hz of *z* axis and measures the frequency between −0.3 and 0.3 Hz to indicate which direction the vehicle is leaning, forward or backward.

Third, the curvature of the road on which the vehicle is currently driving, the speed bump section of the road, etc. are calculated to remove the pressure data irrelevant to the roughness of the road. The RRCS is applied to the KNN by determining the curvature of the driving road and speed bumps based on the braking information on the map. Fourth, the roughness of the road is classified into eight levels by using the data of Nos. 1 to 11 in Table 1 as an input data set. Nos. 12 to 19 in Table 1 indicate the eight-level labels classified by the KNN.

#### 3.2.2. A Design of the BPCS

The BPCS proposed in this paper calculates the brake pressure optimized for driving by using the roughness of the road classified in the RRCS, the condition of surrounding vehicles, and the remaining distance to the destination. Since the BPCS only calculates brake pressure regardless of direction, real time and accuracy can be guaranteed with little input. Table 2 shows the input data sets used for the BPCS.

Since it is difficult to generate abnormal values in the input data set during autonomous driving, and the generated abnormal values should not be ignored, the BPCS normalizes the input data to between 0 and 1 using min-max normalization. Equation (2) represents the min-max normalization.

(2) $$x_{i}^{\prime }=\frac{x_{i}-\min\left(x\right)}{\max\left(x\right)-\min\left(x\right)}$$

In Equation (2), min and max mean the maximum and minimum values that the corresponding data can have, not the maximum value among each item of data. In Table 2, all input data except for the friction coefficient indicated in No. 10 were normalized through min-max normalization. The friction coefficient was determined by the current weather and the speed of the vehicle. Table 3 shows the friction coefficient used for the BPCS.

In Table 2, $d_{brack}$ was calculated using the friction coefficient. Equation (3) shows that the $d_{brack}$ value was calculated by the RPCS.

(3) $$d_{brack}=\frac{v^{2}}{2g\mu },$$

In Equation (3), $d_{brack}$ means the braking distance, $v^{2}$ means the current speed of the vehicle, g means the acceleration of the vehicle, and μ means the friction coefficient between the ground and the tire. Since the BPCS does not know the braking pressure before calculating the braking pressure, $d_{brack}$ means the braking distance when the braking pressure is at its maximum. BPCS uses the normalized data in Table 2 as the node value of the input layer and outputs a value between 0 and 1. That is, the output layer of the BPCS uses one node. Equation (4) shows the output node of the BPCS. In the output of the BPCS, 0 means no braking required for driving and 1 means the maximum use of braking pressure.

(4) Y={y | 0≤y≤1},

The BPCS uses the swish function and the sigmoid function designed by Google to solve the vanishing gradient problem of the existing neural network and fix the value of the output node between 0 and 1. The swish function is used to calculate the value of the node up to the input layer and the last hidden layer, and the sigmoid function is used to calculate the value of the node *y* of the output layer. Equation (5) represents the swish function. Equation (6) represents the sigmoid function. Equation (7) represents the differential function of the swish function for gradient descent. Equation (8) represents the sigmoid differential function.

(5) $$sh\left(x\right)=\frac{1}{1+e^{-x}}x,$$

(6) $$sg\left(x\right)=\frac{1}{1+e^{-x}},$$

(7) $${sh}^{\prime }=sh\left(x\right)+sg\left(x\right)\left(1-sh\left(x\right)\right),$$

(8) $${sg}^{\prime }\left(x\right)=sg\left(x\right)\left(1-sg\left(x\right)\right),$$

The cross entropy error (CEE) has higher accuracy than the root mean squared error (RMSE) when selecting one of several output nodes as the correct answer, but the BPCS has only one output node [30]. The value of the output node is important; so, the RMSE is used as a loss function. Equation (9) shows a computation method of the RMSE.

(9) $$\mathrm{RMSE}=\sqrt{{\left(y-t\right)}^{2}},$$

**Figure 3:**
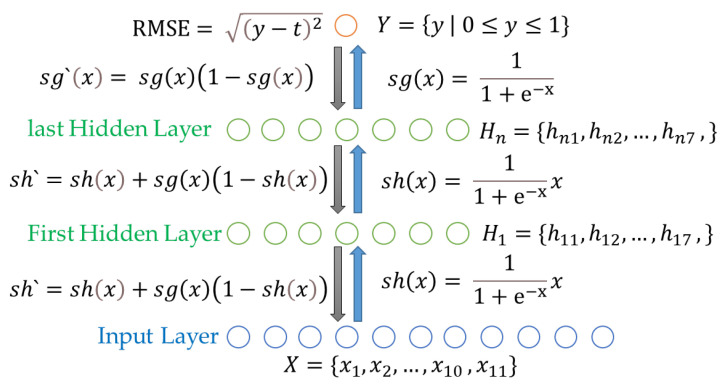
The structure of the BPCS.

In the existing neural network, the RMSE computes the final error by adding the error values of all output nodes; but, since the BPCS has only one output node, the error signal can be calculated simply. The hidden layer of the BPCS consists of seven nodes. The number of hidden layers is determined through experimentation. Figure 3 shows the overall structure of the BPCS.

### 3.3. The Passenger Recognition and Sharing Module (PRSM)

**Figure 4:**
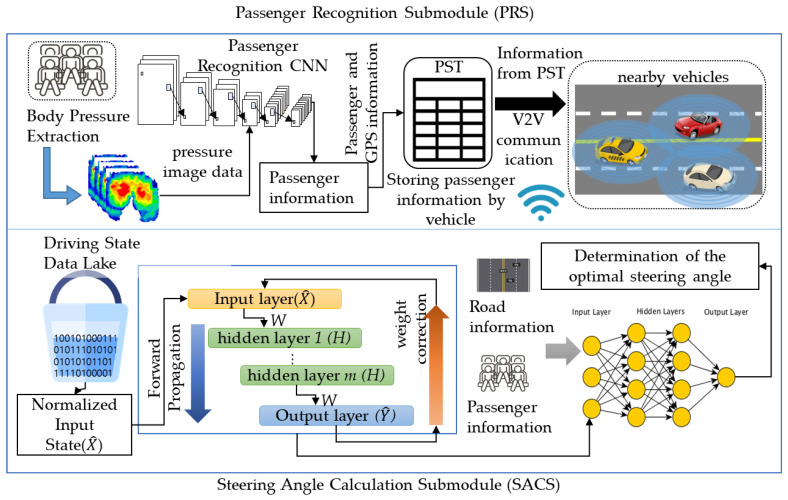
The structure of the PRSM.

The passenger condition of the vehicle must always be considered for the safety of the vehicle. In particular, fully autonomous vehicles need to calculate optimal driving not only for normal driving but also for emergency situations. In such an emergency situation, the vehicle should set a direction to minimize human casualties; it is very important to understand the passenger status of the surrounding vehicles to minimize human casualties [31,32]. Therefore, for this paper, we designed the passenger recognition and sharing module (PRSM), which calculates the optimal steering angle that can minimize damage in emergency situations by detecting the passenger status of the driving vehicle itself and surrounding vehicles. The PRSM consists of two submodules. The passenger recognition submodule (PRS) generates a training data set using generative adversarial networks (GAN) [33] based on the data of people directly seated on the body pressure sensor and designs a passenger recognition CNN (PRCNN) to identify luggage, adults, and children. The steering angle calculation submodule (SACS) computes the optimal steering angle while driving according to the passenger status, driving route, and driving situation of surrounding vehicles. Figure 4 shows the overall concept of the PRSM.

#### 3.3.1. A Design of the PRS

Existing vehicles identify whether a seat is occupied based on the weight applied to the seat. The PRS proposed in this paper collects seat pressure data using a 64*64 body pressure sensor and uses CNN to classify whether existing in the seat is an adult, a child, or an object. Next, the PRS stores the seat information and the location of your vehicle and shares them with the surrounding vehicles. First, sufficient body pressure sensor data are required for PRS learning. Using 20 men and 12 women in their 20s and 30s, 7 men and 3 women in their 50s and 60s, and 7 male children aged 8 to 10, we collect the body pressure sensor data from nine boxes of 10 to 60 kg; the training data for the CNN were generated using a generative adversarial network (GAN) based on the corresponding data.

**Figure 5:**
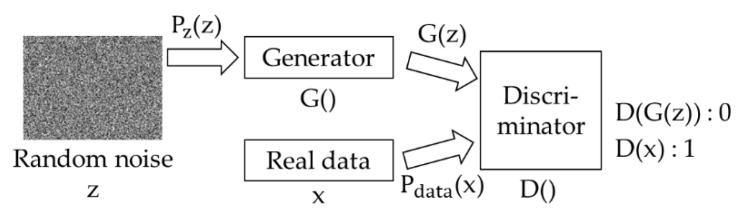
The simple structure of the GAN.

The GAN is an artificial intelligence model that uses unsupervised learning to generate image data. Figure 5 shows the simple configuration of the GAN. In Figure 5, the generator generates new image data using the samples P_z_(z) and G(z) extracted from noise and delivers the generated image G(z) to the discriminator. The purpose of the discriminator is to determine that G(z) is a fake image (that is, to output 0) and determine that P_data_(x) is a real image (that is, to output 1). The generator and discriminator are each a neural network model. Since the GAN is an unsupervised learning model, it learns using the entropy-based loss function rather than the label. When training is completed, the GAN creates a new image. Equation (10) shows the loss function of the GAN.

(10) $$\underset{G}{\min}\;\underset{D}{\max}\;V\left(G,\;D\right)=E_{X∼\mathrm{Pdata}\left(x\right)}\left[\mathrm{logD}\left(x\right)\right]+E_{z∼p_{z}\left(z\right)}\left[\log\left(1-D\left(G\left(z\right)\right)\right)\right],$$

**Figure 6:**
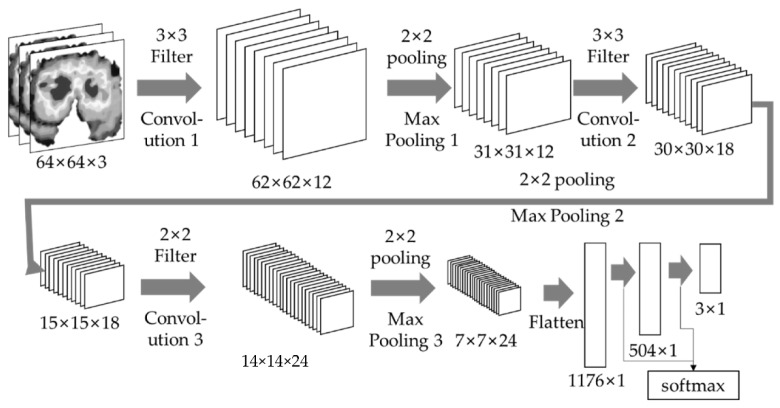
The structure of the PRS.

In the GAN, the learning proceeds in the direction where $V\left(G,\;D\right)$ becomes the maximum while $E_{z∼p_{z}\left(z\right)}\left[\log\left(1-D\left(G\left(z\right)\right)\right)\right]$ becomes the minimum. When the training of the GAN was completed, our work generated 150 images based on 58 images. At this time, the PRS trains the CNN model using these 208 images. The PRS classifies 64 × 64 images into three, R, G, and B, channels, uses 64 × 64 × 3 image data as input data, and decides whether it is an adult seat, a child seat, or cargo seat image. The PRS conducts a convolution and max pooling process three times. Since the input image is small, the stride of the PRS is explained as 1 to ensure accuracy. The PRS uses a 3 × 3 size filter in the first and second convolution processes and uses a 2 × 2 filter in the third convolution process. The first convolution process uses 12 filters, the second convolution process uses 18 filters, and the third convolution process uses 24 filters. The last layer of CNN, the fully connected layer, consists of a neural network with one hidden layer. Figure 6 shows the overall structure of the CNN used in the PRS. The PRS recognizes the current seat state through the CNN and creates a passenger state table (PST) that stores the overall seat state of the vehicle. The PRS stores the current vehicle seat status and vehicle location in the PST and transmits the PST information to the surrounding vehicles through V2V communication. The PRS represents the information of up to 15 seats in an array. Algorithm 1 shows that the PRS stores the seat information in the array and sends it to the PST.
**Algorithm 1. Information stored in PST**Input: Status of each seatOutput: Data set to pass to PST1. Initialize an empty array P[15]2. **FOR** each seat i in range (length (status of seats))  2.1. **IF** is it an adult to exist in seat i?    2.1.1. P[i] = 0  2.2. **IF** is it a child that exists in seat i?    2.2.1. P[i] = 1  2.3. **IF** is it a cargo to exist in seat i?    2.3.1. P[i] = 2  2.4. **IF** is it empty in seat i?    2.4.1. P[i] = 33. **FOR** last i in range (15)3.1 P[i] = 44. Pass P[15] and GPS latitude and longitude to PST

In Algorithm 1, the PRS stores adults as 0, children as 1, cargo as 2, and empty seats as 3 in P[15], and stores GPS information and P[15] in the PST by padding the remaining seats with 4. For example, if a five-seat vehicle has an adult in the driver’s seat, cargo in the passenger’s seat, and a child in the right rear seat, the array of P[15] is created as 023314444444444 and it is stored in the PST. Table 4 shows examples of information stored in the PST. In Table 4, the ‘own’ field indicates whether the car in which the information is stored is a user’s or a surrounding vehicle.

#### 3.3.2. A Design of the SACS

The SACS proposed in this paper computes the optimal steering angle while driving using the passenger status of the owner and the surrounding vehicles, current driving status, and direction to the destination, etc., which are recognized by the PRS. The PRS uses an input data set that is almost similar to the BPCS but, because the BPCS does not consider braking, it uses an input data set that is more focused on the direction of the vehicle than the BPCS. Table 5 shows the input data sets used for the SACS.

Unlike the BPCS, the SACS does not consider acceleration per unit time and uses the distance to the nearest left and right vehicles as input for steering accuracy. It also uses the current steering angle and the curvature of the road to prevent departure from driving due to steering angle changes. The SACS uses the same swish function as the BPCS from the input layer to the last hidden layer but uses the tanh function for the activation function between the last hidden layer and the output layer for the accuracy of result interpretation and learning. The tanh function has a value between −1 and 1, and the SACS computes −1 as right 45° and 1 as left 45° and outputs the final steering angle. Equation (11) shows the tanh function used by SACS, and Equation (12) shows the differential function of the tanh function.

(11) $$\tanh\left(x\right)=\frac{e^{x}-e^{-x}}{e^{x}+e^{-x}},$$

(12) $$\tanh^{\prime }\;\left(x\right)=1-\tanh^{2}\left(x\right),$$

## 4. Simulations

For this paper, four simulations were conducted to verify the efficiency of the BDDS. The simulation environment shown in Table 6 was used for this paper and all machine learning models were made in the Python language using TensorFlow. The simulation was conducted using virtual data. Berkeley DeepDrive’s data set was used to understand the vehicle’s surrounding environment and the driving conditions of the surrounding vehicles. Data not included in Berkeley DeepDrive, such as roughness of roads and distances to destinations, were generated in a virtual environment by our workstation and ECU model. The simulation of this paper was conducted in a virtual environment and was as follows.

- In order to find K suitable for the RRCS, this work computed the error of the RRCS while increasing K from 1 to 31.
- To verify the efficiency of the RRCS, the K-means clustering algorithm and the road classification accuracy of the RRCS were compared.
- This work examined the accuracy of the test data while increasing the number of hidden layers from 1 to 30 in order to obtain the number of hidden layers suitable for the BPCS.
- To verify the accuracy of the PRS, that of the PRS and that of the passenger recognition system of the existing vehicle were compared.
- Data from 1204 traffic accidents in Korea were used to measure the efficiency of braking pressure and passenger detection. The speed and steering angle at the time of the vehicle accident, the output of the BDDS excluding the roughness of the road and the passenger condition, and the output of the BDDS including the roughness of the road and the passenger condition were compared.

### 4.1. Simulations of the RRCS

**Figure 7:**
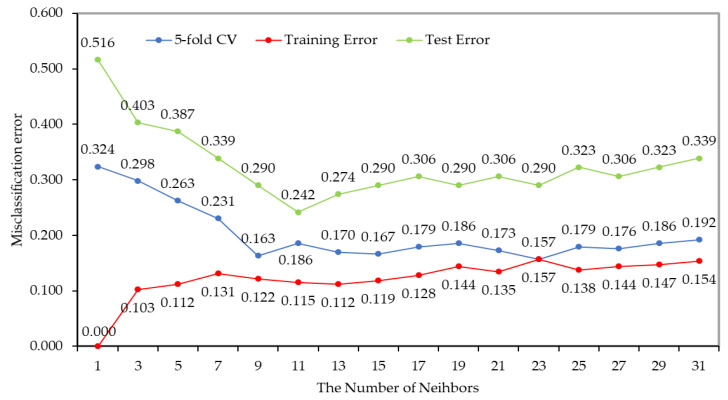
The misclassification errors of the RRCS.

First, this work conducted a simulation to find K suitable for the RRCS. For the simulation, 312 data sets were used, of which 62 sets were used as test data sets and the remaining 250 sets were subjected to five-fold cross validation. Figure 7 shows the misclassification errors of the RRCS.

The simulation result showed that the RRCS had the lowest classification error when the K of KNN was used as 11. Additionally, there was no significant difference in the classification error from 11 to 21, but when K was greater than 23, the classification error was increased due to overfitting. Therefore, it is most appropriate to use K as 11 for the RRCS.

**Figure 8:**
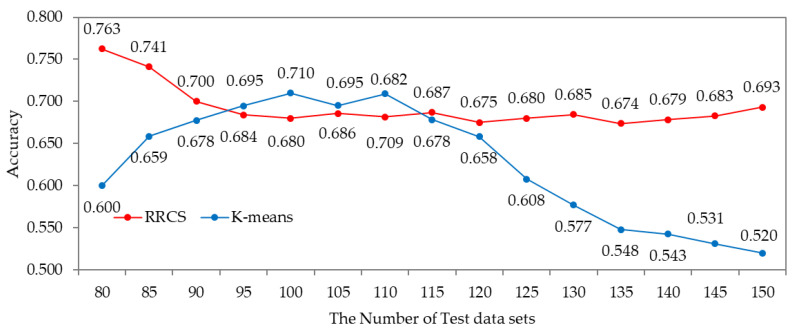
The accuracy of the RRPS and K-means algorithms.

Second, to verify the accuracy of the RRCS, the accuracy of the K-means clustering algorithm trained with the same training data and that of the RRCS were compared. The RRCS was trained using 200 training data sets, and the RRCS and K-means algorithms were tested using 150 test data sets. Figure 8 shows the accuracy when the test data sets are input to the RRPS and K-means clustering algorithms. According to the result of the simulation, the average accuracy of the RRCS was 0.693 and that of the K-means algorithm was 0.627. Because the K-means algorithm classified unlabeled data into eight clusters, it showed higher accuracy than the RRCS when classifying a constant amount of data but failed to classify between clusters as the amount of data increased. Therefore, the RRCS is more suitable than K-means algorithm to classify road roughness.

### 4.2. Simulations of the BPCS

**Figure 9:**
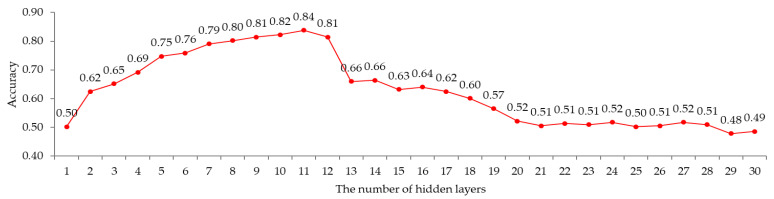
The accuracy of the RRPS and K-means.

Next, this work examines the accuracy of the BPCS according to the number of hidden layers in order to obtain the number of hidden layers suitable for the BPCS. In this paper, the BPCS was trained using 253 training data sets while fixing the number of nodes of the hidden layer to seven and increasing the number of hidden layers of the BPCS from 1 to 30. Figure 9 shows the accuracy of the BPCS according to the number of hidden layers.

The result of the simulation showed that, when the number of hidden layers increased to 11, the accuracy of the BPCS increased to 84%, but when the number of hidden layers was increased to 13, the accuracy decreased sharply. Therefore, it is most suitable for the BPCS to use 11 hidden layers.

### 4.3. Simulations of the PRS

This work compared the accuracy of the existing passenger recognition system and the PRS to verify the efficiency of the PRS. The existing passenger recognition system recognized accurately in the following cases.

- To correctly recognize the person seated in the seat as a person, whether an adult or a child;
- To recognize a seat loaded with cargo as an empty seat.

**Figure 10:**
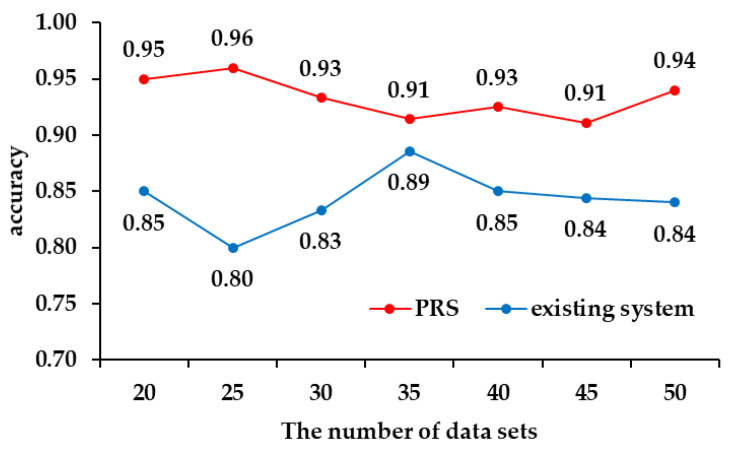
The accuracy of the PRS and the existing recognition system.

For the simulation, it was carried out by increasing the data set from 20 to 50, and the proportion of the cargo was increased each time the amount of data was increased. Table 7 shows the number of pieces of cargo included in the data set, and Figure 10 shows the accuracy of the PRS and the existing system for each data set.

As a result of the simulation, the PRS showed that it had about 9% higher accuracy than the passenger recognition system of the existing vehicle. The accuracy of the PRS was measured as high with a small amount of training data and simulation data, but most of the difference in accuracy occurred in the recognition of 10 kg to 30 kg cargo. Thus, the PRS can distinguish cargo better than the existing passenger recognition systems.

### 4.4. Simulations of the BDDS

**Figure 11:**
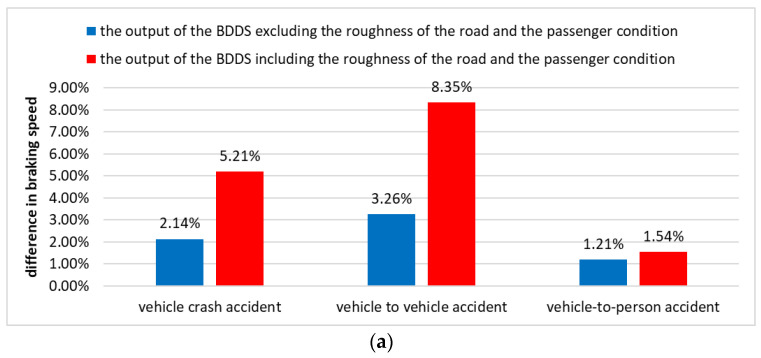

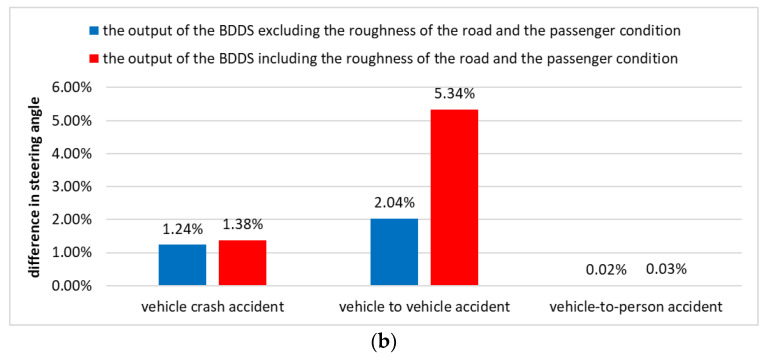
(**a**) Difference between accident data and BDDS results (braking speed). (**b**) Difference between accident data and BDDS results (steering angle).

Finally, in order to verify the effectiveness of the BDDS, this work compared the actual accident data, the output of the BDDS without road roughness and passenger status, and the output of the BDDS with road roughness and passenger status. This experiment represented the average of the difference between the output of BDDS and the actual accident data in 171 vehicle–object accidents, 483 vehicle–vehicle accidents, and 550 vehicle–person accident items of data. The actual data used for the simulation contained at least one death. Figure 11a shows the difference in braking speed according to road roughness and passenger conditions, and Figure 11b shows the difference in steering angle.

Figure 11 shows the difference between the driving condition of the vehicle and the output of the BDDS at the time of the accident as a percentage. As a result of the simulation, the BDDS requires much more braking when the vehicle collides with a large object (or vehicle). In addition, the BDDS showed a much greater difference in steering angle when a vehicle-to-vehicle collision occurred than in a conventional accident. Therefore, the BDDS can determine the driving method more aggressively than the existing vehicle driving method in order to increase stability in crisis situations and prevent death.

## 5. Conclusions

This paper proposed that a braking-pressure and driving-direction determination system (BDDS) consists of the road roughness classification module (RRCM) and passenger recognition and sharing module (PRSM). The RRCM classified road roughness based on a KNN algorithm, and the PRSM accurately recognized passengers inside the vehicle based on a CNN and shared it with surrounding vehicles. In the simulation, this work found the optimal parameters required for the AI model and proved the efficiency of each module. As a result of the simulation, the RRCS to classify the roughness of the road had about 7% higher accuracy than the K-means clustering algorithm, and the PRS to recognize passengers had 9% higher accuracy than the existing vehicle passenger recognition system. Therefore, the expected effects of the BDDS are as follows.

- The BDDS can extend the recognition range of existing autonomous vehicles that rely heavily on visual data (from lidar, front camera, etc.).
- Because BDDS modularized brake pressure and steering angle computations, each AI model is accurate and simple.

In this paper, limited deep learning models using about 400 data sets were trained. Future research should further train and test the BDDS by increasing the data sets. Since the seat recognition sensor of the existing vehicle cannot be simulated in a virtual environment, the comparison between the PRS and the existing vehicle was conducted with little data. Future research should implement the vehicle seat recognition system in a virtual environment and conduct simulations with more data. Additionally, if experiments with real vehicles become possible and research advances enough to determine braking and steering except for computer vision, the BDDS could also be used in the development of ADAS for special vehicles (such as forklift trucks) that are not provided with autonomous driving systems [34,35].

## Figures and Tables

**Table 1:** Input data set and clusters the RRCS.

No.	Variable Name	Destination
1	cs	Current speed
2	Δs	Acceleration for 3 s
3	a	Steering angle
4	$p_{lt}$	Pressure (left, top)
5	$p_{rt}$	Pressure (right, top)
6	$p_{lb}$	Pressure (left, bottom)
7	$p_{rb}$	Pressure (right, bottom)
8	b	A speed bump passed in 3 s
9	d	Distance to destination
10	lc	Lane change made in 3 s
11	sl	The slope of the road
12	$C_{1}$	Label 1 of clusters (clean roads)
13	$C_{2}$	Label 2 of clusters (such as highways)
14	$C_{3}$	Label 3 of clusters (paved roads with irregularities)
15	$C_{4}$	Label 4 of clusters (old pavement)
16	$C_{5}$	Label 5 of clusters (roads made of concrete tiles)
17	$C_{6}$	Label 6 of clusters (A road made of bricks such as tiles)
18	$C_{7}$	Label 7 of clusters (dirt road)
19	$C_{8}$	Label 8 of clusters (off road)

**Table 2:** Input data set of the BPCS.

No.	Variable Name	Destination
1	cs	Current speed
2	Δs	Acceleration for 3 s
3	$d_{dest}$	Distance to destination
4	Δlc	Lane change made in 3 s
5	sl	The slope of the road
6	$f_{d}$	Distance from the vehicle in front
7	$f_{a}$	Relative acceleration with the vehicle in front for 3 s
8	p_lc	Whether to change lanes within 3 s
9	r	Roughness of the road
10	cf	Coefficient of friction
11	$d_{brack}$	Estimated braking distance

**Table 3:** Context-specific friction coefficient.

Road and Driving Conditions		Pavement Road	Cracked Road	Unpaved Road
Dry road	48 km/h or more	0.45~0.70	0.35~0.60	0.40~0.70
	Less than 48 km/h	0.55~0.80	0.50~0.60	0.40~0.70
Wet road	48 km/h or more	0.45~0.65	0.25~0.55	0.45~0.75
	Less than 48 km/h	0.45~0.70	0.30~0.60	0.45~0.75

**Table 4:** An example of the PST.

No.	Own	GPS	Passenger
1	1	42.10321, 2.17403	001014444444444
2	0	42.10338, 2.17403	003333334444444
…	…	…	…
n	0	42.22457, 2.17403	001012222222222

**Table 5:** Input data set of the SACS.

No.	Variable Name	Destination
1	cs	Current speed
2	Δlc	Lane change made in 3 s
3	$f_{l}$	Distance from the vehicle in left
4	$f_{r}$	Distance from the vehicle in right
5	$p_{lc}$	Whether to change lanes within 3 s
6	cf	Coefficient of friction
7	$P_{tr}$	Whether to turn left within 10 s
8	$p_{tl}$	Whether to turn right within 10 s
9	cv	Curvature of the road
10	ca	Current steering angle

**Table 6:** The hardware environment used for simulation.

Part Name	Specification
CPU	Intel i5-6500 3.20 GHz
RAM	DDR4 16 GB
GPU	GTX1060 VRAM 6 GB
SSD	125 GB

**Table 7:** The input data set of the SACS.

The Number of Total Data Sets	5 kg Cargo Data (EA)	10 kg Cargo Data (EA)	20 kg Cargo Data (EA)	30 kg Cargo Data (EA)
20	2	1	1	1
25	2	2	1	1
30	2	2	1	1
35	2	2	2	1
40	2	2	2	1
45	2	2	2	2
50	3	2	2	2
